# Investigation on regulation of *N*-acetyltransferase 2 expression by nuclear receptors in human hepatocytes

**DOI:** 10.3389/fphar.2024.1488367

**Published:** 2024-11-18

**Authors:** Kyung U. Hong, Anthony P. Aureliano, Kennedy M. Walls, David W. Hein

**Affiliations:** ^1^ Department of Pharmacology and Toxicology and Brown Cancer Center, University of Louisville School of Medicine, Louisville, KY, United States; ^2^ Department of Pharmaceutical and Administrative Sciences, College of Pharmacy and Health Sciences, Western New England University, Springfield, MA, United States

**Keywords:** N-acetyltransferase 2, NAT2, hepatocytes, nuclear receptors, FXR, PPARα, PXR, LXR

## Abstract

**Introduction:**

Arylamine *N*-acetyltransferase 2 (NAT2) expresses a well-defined genetic polymorphism in humans that modifies drug and xenobiotic metabolism. Recent studies and genome wide association studies have reported that genetic variants of *NAT2* are associated with differential risks of developing dyslipidemia and cardiometabolic disorders, suggesting a previously unrecognized role of NAT2 in pathophysiology of metabolic disorders. In support of this notion, we recently showed that human *NAT2* expression is differentially regulated by glucose and insulin. Moreover, our *in silico* analysis showed that *NAT2* is co-expressed with nuclear receptors enriched in the liver, e.g., *NR1H4* (FXR) and *NR1I2* (PXR), that have been previously implicated in regulation of hepatic glucose and lipid homeostasis. Identification of transcriptional regulator(s) of human *NAT2* would aid in understanding novel functions that it may play in the liver. Thus, the present study was designed to investigate if *NAT2* is transcriptionally regulated by hepatic nuclear receptors.

**Methods:**

To test this, we treated cryopreserved human hepatocytes with agonists towards four different hepatic transcription factors/nuclear hormone receptors, namely FXR (NR1H4), PXR (NR1I2), LXR (NR1H3), and PPARα (PPARA), and measured their effects on the level of *NAT2* mRNA.

**Results:**

While the treatment with a FXR, PXR, or LXR agonist (i.e., GW-4064, SR-12813, or GW-3965) significantly induced their respective target genes, treatment with these agonists did not significantly alter the transcript level of *NAT2* in human hepatocytes. PPARα agonist, GW-7647, treatment resulted in a statistically significant decrease in the *NAT2* transcript level. However, its magnitude was marginal.

**Conclusion:**

In summary, hepatic nuclear receptors we examined in the present study (FXR, PXR, LXR, and PPARα) did not significantly alter *NAT2* expression in cryopreserved human hepatocytes. Additional studies are needed to identify transcriptional regulators of hepatic NAT2 expression.

## 1 Introduction

Arylamine *N*-acetyltransferase 2 (NAT2) is a phase II metabolic enzyme commonly known for its role in biotransformation of aromatic amines and hydrazines as reviewed previously (McDonagh et al., 2014). *NAT2* expresses well-defined genetic polymorphisms, and previous studies have shown that single nucleotide polymorphisms (SNPs) in the *NAT2* coding region often lead to changes in protein stability or altered substrate affinity of NAT2 (Hein, 2002). Combination of variant *NAT2* alleles manifest as three different acetylator phenotypes in the general population based on the relative activity level they produce: slow, intermediate, and rapid acetylators (Hein, 2002; Sim et al., 2014). The acetylator phenotype has a profound impact on the rate of certain drug (e.g., isoniazid and hydralazine) and carcinogen (e.g., 4-aminobiphenyl) metabolism in individuals (Hein and Millner, 2021).

In recent years, studies have suggested that NAT2 plays unexpected roles in human pathophysiology. In a genome wide association study (GWAS), *NAT2* coding SNPs, rs1208 and rs1801280, has been associated with insulin resistance (assessed by the euglycemic clamp method), independent of body-mass index (Knowles et al., 2015). Follow up studies reported that *Nat1* (the functional ortholog of human *NAT2*) knockout mice exhibit multiple signs of metabolic dysfunction, including elevated fasting blood glucose, insulin resistance, mitochondrial dysfunction, decreased fat utilization, and marked increases in tissue fat content (Chennamsetty et al., 2016; Camporez et al., 2017), supporting the role of murine NAT1 in insulin sensitivity and energy utilization *in vivo*. Although the mechanism remains obscure, these studies indicate that variable levels (or activities) of NAT2 may influence the state of energy storage and utilization.

Recent reports from our group implicated NAT2 in regulation of lipid/cholesterol homeostasis in the liver. *NAT2* transcript is upregulated by glucose and insulin in liver cancer cell lines (Hong et al., 2022), suggesting that its expression is regulated by the nutrient status. Moreover, according to our *in silico* analysis, human *NAT2* is co-expressed with the genes involved in lipid and cholesterol synthesis and transport, such as *APOB, ABCG8, ANGPTL3, FABP1, MOGAT2,* and *PLA2G12B* (Hong et al., 2022). Numerous GWAS reports also implicate NAT2 in regulation of plasma lipid and cholesterol levels (Hong et al., 2023), as multiple *NAT2* genetic variants have been associated with differential plasma lipid and cholesterol levels. Interestingly, the risk alleles for dyslipidemia of *NAT2* gene (e.g., rs1495741-A) are associated with the rapid acetylator phenotype in humans (Hong et al., 2023), which suggests that individuals with increased levels of NAT2 activity are at a higher risk of developing dyslipidemia. The link between NAT2 acetylator phenotype and plasma lipid levels is also supported by our previous study on rats congenic for rapid or slow *Nat2* acetylator genotypes. Regardless of the diet, rapid acetylator rats were more prone to develop dyslipidemia (i.e., higher triglyceride; higher LDL; and lower HDL), compared to slow acetylator rats (Hong et al., 2020).

Taken together, it appears that the level of NAT2 is not only regulated by the nutrient status (e.g., glucose and insulin) but also may play an important role in regulating lipid/cholesterol homeostasis, presumably in liver and intestines which are two tissues with relatively high expression. Although many studies have examined the functional outcomes of genetic variants of *NAT2* in the context of xenobiotic metabolism, so far, few studies have explored its transcriptional regulation (Zou et al., 2020; Zhu et al., 2021; Hong et al., 2022). In our previous study, we reported that the genes that are co-expressed with *NAT2* include those encoding hepatic nuclear receptors, e.g., farnesoid X receptor (FXR; NR1H4) and pregnane X receptor (PXR; NR1I2). Nuclear receptors function as ligand-activated transcription factors (Frigo et al., 2021). For example, PXR is commonly known to transactivate genes that encode proteins involved in xenobiotic metabolism, but also plays a role in glucose homeostasis and insulin sensitivity (Kliewer et al., 2002; Spruiell et al., 2014). FXR, liver X receptor alpha and beta (LXRα/β), and peroxisome proliferator activated receptor alpha (PPARα) respond to changes in cellular levels of endogenous lipid ligands by regulating the expression of genes involved in lipid metabolism (Calkin and Tontonoz, 2012; Hong and Tontonoz, 2014). This prompted us to investigate the mechanisms of transcriptional regulation of *NAT2* by nuclear receptors expressed in the liver.

In the present study, we tested if FXR, PXR, LXR, or PPARα regulate *NAT2* expression. We treated cryopreserved human hepatocytes with specific agonists for the nuclear receptors and compared the transcript levels of *NAT2* as well as known target genes of the receptors.

## 2 Materials and methods

### 2.1 Cell culture

Cryopreserved human hepatocytes were purchased from BioIVT (http://www.bioivt.com) and stored in liquid nitrogen until use. The hepatocytes were thawed and cultured in an incubator with a humidified air (95%) and CO_2_ (5%) at 37°C as previously reported (Walls et al., 2023). Briefly, cells were thawed by warming at 37°C for 90 s and suspending them in InVitroGRO HT medium (BioIVT) containing TORPEDO™ Antibiotic Mix (BioIVT) and plated on Biocoat^®^ collagen-coated plates (Corning).

### 2.2 Agonists for nuclear receptors

GW-4064 (FXR agonist), SR-12813 (PXR agonist), GW-3965 (LXR agonist), and GW-7647 (PPARα agonist) were purchased from Selleck. The working solutions were prepared in DMSO. The final treatment concentrations were the following: 1 μM GW-4064, 1 μM SR-12813, 2 μM GW-3965, and 10 μM GW-7647. The cryopreserved human hepatocytes, in replicates of three, were treated with DMSO (vehicle control) or an agonist for 48 h prior to the harvest.

### 2.3 Gene expression analysis

Following the agonist treatment, total RNA was isolated from the control and the treated cells using E.Z.N.A. Total RNA Kit 1 (Omegabiotek) according to the manufacturer’s instructions. cDNA synthesis was done using High-Capacity cDNA Reverse Transcriptase PCR kit (Thermo Scientific). Gene-specific cDNA was amplified and detected using iTaq Universal SYBR Green Supermix (Bio-Rad), StepOne real-time PCR system (Applied Biosystems), and gene-specific primers (see Table 1 for primer sequences). Results were normalized to an internal control gene, *GAPDH*, and the relative fold change was calculated using the delta-delta Ct (2^−ΔΔCT^) method. The statistical analyses were performed using GraphPad Prism v8.2.1 (GraphPad Software). Unpaired *t*-test was employed to compare between the control and treatment groups. The results are expressed as the mean ± standard error of the mean (SEM) and from three independent experiments (n = 3). Statistical significance was determined per the following *p*-values: *, *p* < 0.05, **, *p* < 0.01, ***, *p* < 0.001, ****, *p* < 0.0001.

**TABLE 1:** List of PCR primer sequences used in the study.

Gene (human)	Description		Sequence
***ABCA1***	ATP binding cassette subfamily A member 1	Forward	GCT​GGT​GTG​GAC​CCT​TAC​TC
		Reverse	GCA​GCT​TCA​TAT​GGC​AGC​AC
***ABCB11***	ATP binding cassette subfamily B member 11	Forward	AAC​AGG​CTC​AGC​TGC​ATG​AT
		Reverse	CTG​GAT​GGT​GGA​CAA​GCG​AT
***ABCG5***	ATP binding cassette subfamily G member 5	Forward	CTC​GCA​GGA​ACC​GAA​TTG​TG
		Reverse	GGC​GTG​CCA​CAG​AAA​ATC​AG
***ACOX1***	Acyl-CoA oxidase 1	Forward	GTA​GCA​GTC​TGG​CCA​ACC​AT
		Reverse	GCT​CCC​CTG​AAG​GAA​ATC​CC
***CYP2B6***	Cytochrome P450 family 2 subfamily B member 6	Forward	CCA​CCC​TAA​CAC​CCA​TGA​CC
		Reverse	CCC​AGG​TGT​ACC​GTG​AAG​AC
***CYP3A4***	Cytochrome P450 family 3 subfamily A member 4	Forward	CGG​GAC​TAT​TTC​CAC​CAC​CC
		Reverse	CCC​CAC​GCC​AAC​AGT​GAT​TA
***CYP7A1***	Cytochrome P450 family 7 subfamily A member 1	Forward	AAG​CAA​ACA​CCA​TTC​CAG​CG
		Reverse	CAC​TGG​AAA​GCC​TCA​GCG​AT
***FABP1***	Fatty acid binding protein 1	Forward	GGG​AAG​GGA​GCC​CCC​TAT​AA
		Reverse	TGG​ATC​ACT​TTG​GAC​CCA​GC
***GAPDH***	Glyceraldehyde-3-phosphate dehydrogenase	Forward	GGT​GAA​GCA​GGC​GTC​GGA​GG
		Reverse	GAG​GGC​AAT​GCC​AGC​CCC​AG
***NAT2***	*N*-Acetyltransferase 2	Forward	TGG​ACC​AAA​TCA​GGA​GAG​AGC
		Reverse	GCC​CAC​CAA​ACA​GTA​AAC​CC
***NR0B2***	Nuclear receptor subfamily 0 group b member 2; aka, SHP	Forward	TGC​TGT​CTG​GAG​TCC​TTC​TG
		Reverse	CCA​GGG​TTC​CAG​GAC​TTC​ACA
***SLC2A2***	Solute carrier family 2 member 2; aka, GLUT2	Forward	CCA​GCT​ACC​GAC​AGC​CTA​TT
		Reverse	GGT​TTG​CTG​ATA​CCA​GCC​GT

## 3 Results

### 3.1 Gene expression changes by a FXR agonist, GW-4064

**FIGURE 1:**
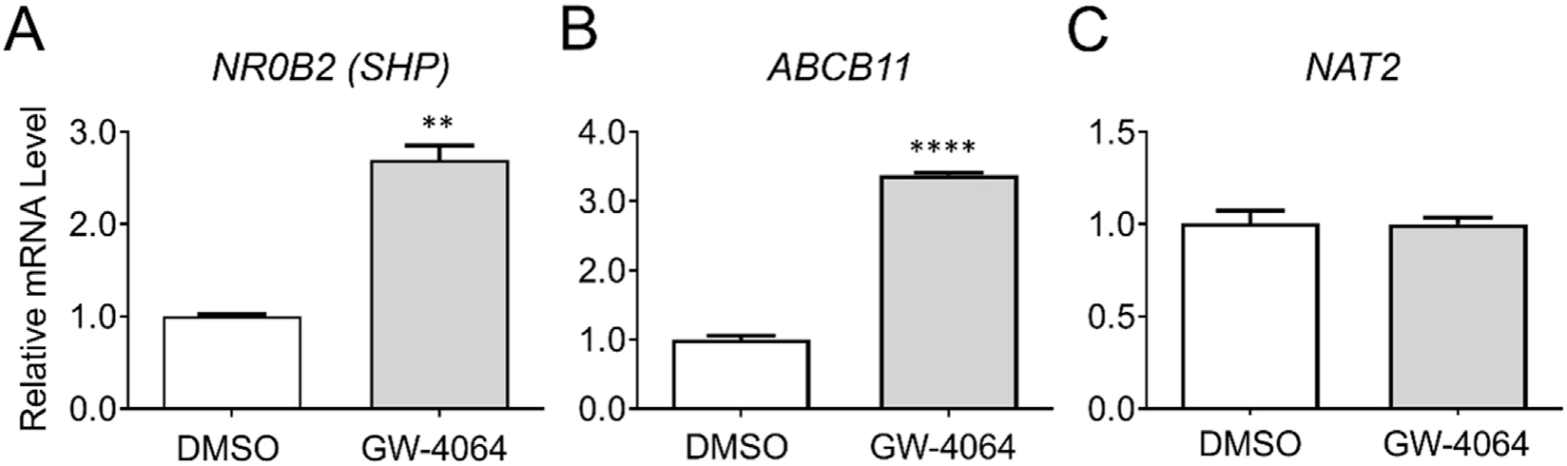
Gene expression changes following activation of FXR. Cryopreserved human hepatocytes were treated with a FXR agonist, GW-4064, for 2 days. The mRNA levels of the indicated genes were measured by RT-qPCR using *GAPDH* as an internal control. The mRNA level in the treated group was then expressed relative to that in the DMSO control. *NR0B2* **(A)** and *ABCB11* **(B)** represent known FXR target genes. GW-4064 did not alter the *NAT2* mRNA level **(C)**. Bars represent mean ± SEM. *, *p* < 0.05; **, *p* < 0.01; ***, *p* < 0.001; ****, *p* < 0.0001.

To evaluate whether *NAT2* is transcriptionally regulated by FXR, we treated cryopreserved human hepatocytes with DMSO as control or a FXR agonist, GW-4064, and measured changes in mRNA expression of *NAT2*. Additionally, we measured changes in the mRNA expression of two known FXR target genes as positive controls: small heterodimer partner [SHP; nuclear receptor subfamily 0 group B member 2 (*NR0B2*)] and ATP binding cassette subfamily B member 11 (*ABCB11*) (Rausch et al., 2022). Expectedly, the FXR agonist treatment resulted in a statistically significant upregulation of both target genes tested [by 2.7-fold for *NR0B2* (*p* < 0.01) and 3.4-fold for *ABCB11* (*p* < 0.0001)], indicating activation of FXR by the agonist (Figures 1A, B). In contrast, the FXR agonist treatment failed to alter *NAT2* mRNA level (Figure 1C), suggesting that *NAT2* is not transcriptionally regulated by FXR in human hepatocytes.

### 3.2 Gene expression changes by a PXR agonist, SR-12813

**FIGURE 2:**
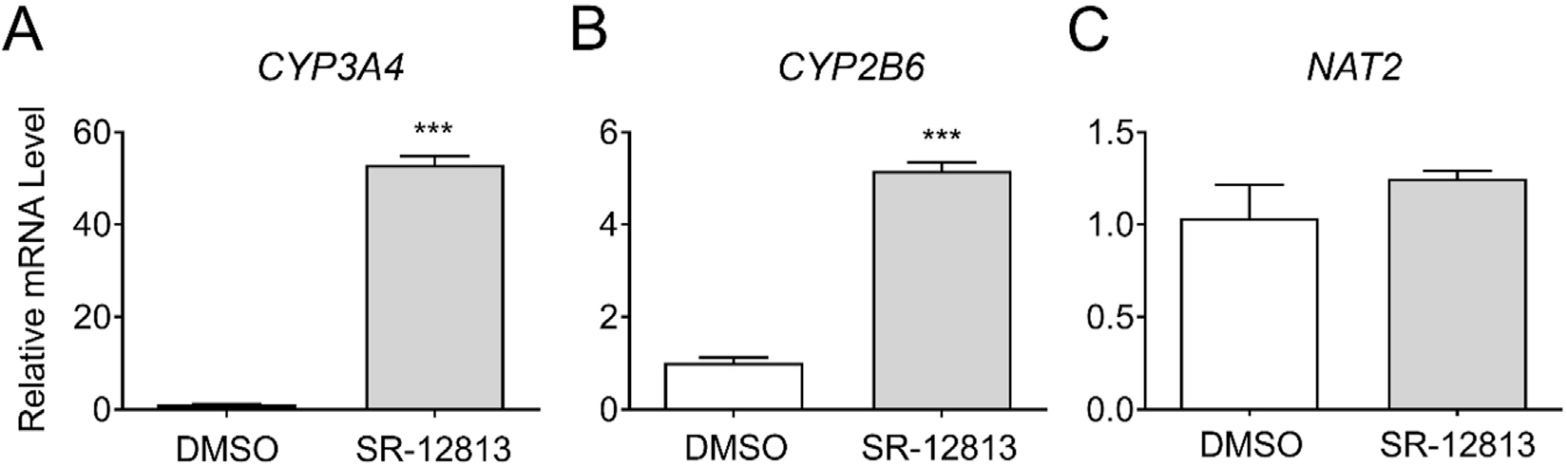
Gene expression changes following activation of PXR. Cryopreserved human hepatocytes were treated with a PXR agonist, SR-12813, for 2 days. The mRNA levels of the indicated genes were measured by RT-qPCR using *GAPDH* as an internal control. The mRNA level in the treated group was then expressed relative to that in the DMSO control. *CYP3A4* **(A)** and *CYP2B6* **(B)** represent known PXR target genes. SR-12813 did not alter the *NAT2* mRNA level **(C)**. Bars represent mean ± SEM. *, *p* < 0.05; **, *p* < 0.01; ***, *p* < 0.001; ****, *p* < 0.0001.

We also assessed if *NAT2* is transcriptionally regulated by PXR. Cryopreserved human hepatocytes were treated with DMSO or a PXR agonist, SR-12813, and changes in the mRNA level of *NAT2* were monitored. Along with *NAT2*, we measured changes in the mRNA expression of two known PXR target genes as positive controls: cytochrome P450 family 3 subfamily A member 4 (*CYP3A4*) and cytochrome P450 family 2 subfamily B member 6 (*CYP2B6*) (Hariparsad et al., 2009). The PXR agonist treatment resulted in a statistically significant upregulation of both target genes tested [by 52.9-fold for *CYP3A4* (*p* < 0.001) and 5.2-fold for *CYP2B6* (*p* < 0.001)], indicating activation of PXR by the agonist (Figures 2A, B). In contrast, the *NAT2* mRNA level was not altered following PXR agonist treatment, suggesting *NAT2* (Figure 2C) is not transcriptionally regulated by PXR in human hepatocytes.

### 3.3 Gene expression changes by a LXR agonist, GW-3965

**FIGURE 3:**
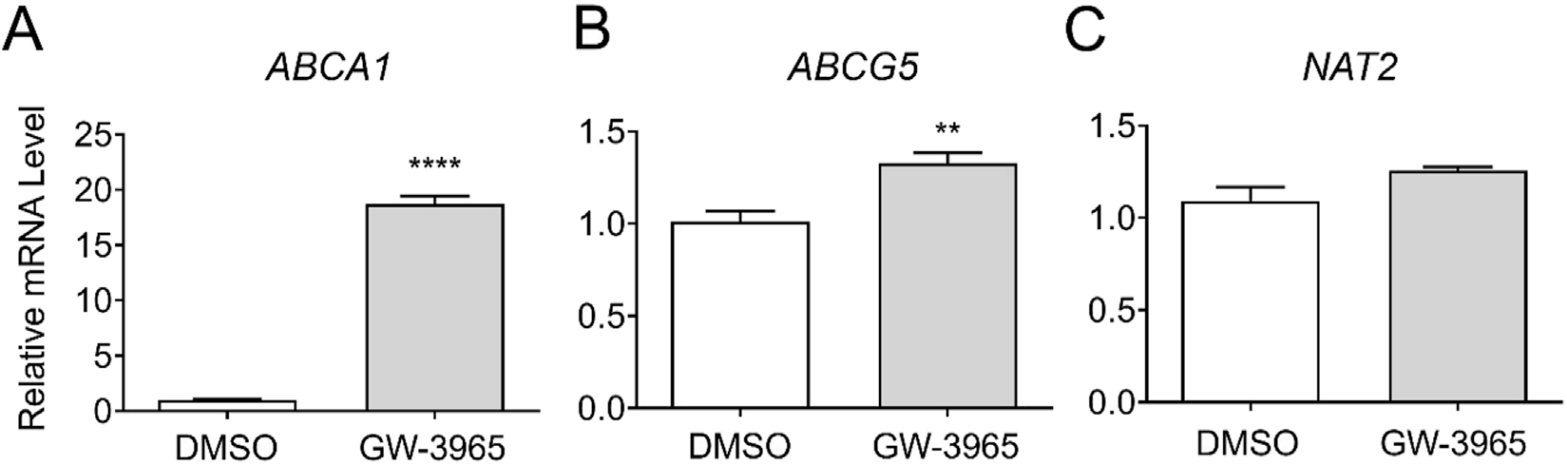
Gene expression changes following activation of LXR. Cryopreserved human hepatocytes were treated with a LXR agonist, GW-3965, for 2 days. The mRNA levels of the indicated genes were measured by RT-qPCR using *GAPDH* as an internal control. The mRNA level in the treated group was then expressed relative to that in the DMSO control. *ABCA1* **(A)** and *ABCG5* **(B)** represent known PXR target genes. SR-12813 did not alter the *NAT2* mRNA level **(C)**. Bars represent mean ± SEM. *, *p* < 0.05; **, *p* < 0.01; ***, *p* < 0.001; ****, *p* < 0.0001.

To investigate the transcriptional regulation of *NAT2* by LXR, we treated cryopreserved human hepatocytes with DMSO or GW-3965, an LXR agonist, and measured changes in *NAT2* mRNA expression and two positive control genes. For positive controls, we used two known target genes of LXR: ATP-binding cassette subfamily A member 1 (*ABCA1*) and ATP binding cassette subfamily G member 5 (*ABCG5*) (Fessler, 2018). While the results of the positive controls were both statistically significant, the LXR agonist treatment of *ABCA1* (18.2-fold increase) had greater statistical significance (*p* < 0.0001) compared to *ABCG5* (*p* < 0.01) (1.3-fold increase) (Figures 3A, B). The *NAT2* mRNA level, however, showed no statistically significant changes after treatment with the LXR agonist, suggesting that *NAT2* is not transcriptionally regulated by LXR in human hepatocytes (Figure 3C).

### 3.4 Gene expression changes by a PPARα agonist, GW-7647

**FIGURE 4:**
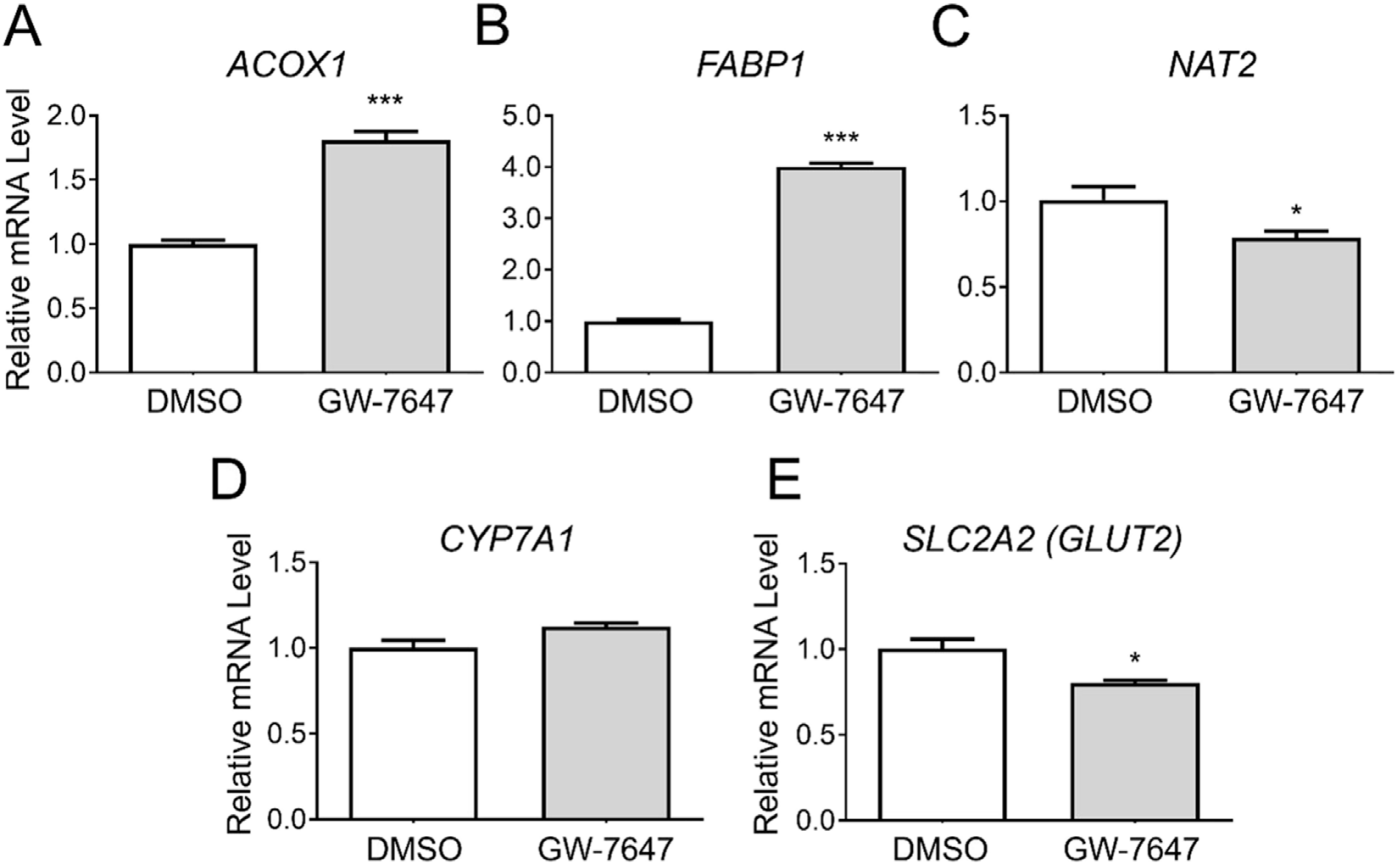
Gene expression changes following activation of PPARα. Cryopreserved human hepatocytes were treated with a PPARα agonist, GW-7647, for 2 days. The mRNA levels of the indicated genes were measured by RT-qPCR using *GAPDH* as an internal control. The mRNA level in the treated group was then expressed relative to that in the DMSO control. *ACOX1* **(A)** and *FABP1* **(B)** were previously shown to be upregulated by PPARα, while *CYP7A1* **(D)** and *GLUT2* **(E)** were reported to be suppressed by PPARα. GW-7647 significantly, yet marginally, decreased the *NAT2* and *GLUT2* mRNA levels **(C, E)**. Bars represent mean ± SEM. *, *p* < 0.05; **, *p* < 0.01; ***, *p* < 0.001; ****, *p* < 0.0001.

To assess whether *NAT2* is transcriptionally regulated by PPARα, we treated cryopreserved human hepatocytes with DMSO or a PPARα agonist, GW-7647. Additional measurements were taken investigating changes in mRNA levels of two known target genes of PPARα that served as positive controls: acyl-CoA oxidase 1 (*ACOX1*) and fatty acid binding protein 1 (*FABP1*) (Rakhshandehroo et al., 2010). Expectedly, the treatment with the PPARα agonist significantly induced the transcript levels of both *ACOX1* and *FABP1* [by 1.8-fold for *ACOX1* (*p* < 0.001) and 4.0-fold for *FABP1* (*p* < 0.001)] (Figures 4A, B). In comparison, *NAT2* showed a statistically significant decrease in the mRNA expression following PPARα agonist treatment (by 0.8-fold) (*p* < 0.05) (Figure 4C). To further validate our data, we chose to include two additional target genes previously shown to be downregulated by PPARα: cytochrome P450 family 7 subfamily A member 1 (*CYP7A1*) and glucose transporter 2 (*GLUT2*) (Rakhshandehroo et al., 2009). The transcript level of *CYP7A1* did not change (Figure 4D), but there was a statistically significant decrease in the mRNA level of *GLUT2* (*SLC2A2*) (by 0.8-fold) (*p* < 0.05) (Figure 4E). Although there was a statistically significant decrease in *NAT2* mRNA expression following PPARα agonist treatment, the slight downregulation of *NAT2* expression may not be biologically significant.

## 4 Discussion

The activation of nuclear receptors, FXR, PXR, and LXR, by corresponding agonists did not result in significant alterations in the *NAT2* mRNA level in cryopreserved human hepatocytes, which suggests that they are not major transcriptional regulators of *NAT2* under the test conditions. However, it is worth noting that activation of PPARα by GW-7647 resulted in a marginal, yet significant, reduction in the *NAT2* transcript level. However, it is not clear if such a minute decrease has any biological impact.

PPARα is activated by endogenous fatty acids and their derivatives (Chakravarthy et al., 2009; Pawlak et al., 2015). PPARα activation promotes fatty acid transport and metabolism (β-oxidation) as well as lipogenesis via transcriptional activation of the genes involved in these processes, including sterol regulatory element binding protein 1c (*SREBP-1c*; *SREBF1*), fatty acid synthase (*FASN*), and acetyl-CoA carboxylase 1 (*ACC1*; *ACACA*) (Patel et al., 2001; Fernández-Alvarez et al., 2011). While *ACOX1* and *FABP1* represented the target genes upregulated by PPARα (Rakhshandehroo et al., 2010), we chose to examine *CYP7A1* and *GLUT2* (*SLC2A2*) as downregulated target genes (see Figure 4). *CYP7A1* encodes cholesterol 7-α-monooxygenase, the first and rate-limiting enzyme in bile acid synthesis from cholesterol (Noshiro and Okuda, 1990). It has been shown to be suppressed by Wy14643, a PPARα agonist, in HepG2 and primary hepatocytes (Marrapodi and Chiang, 2000; Rakhshandehroo et al., 2009). Rakhshandehroo and colleagues reported that the treatment with Wy14643 (another PPARα agonist) results in a significant downregulation of genes involved in glucose absorption, such as glucose transporter 2 (*GLUT2*; *SLC2A2*) and villin-1 (*VIL1*) in both mouse and human primary hepatocytes (Rakhshandehroo et al., 2009). In our study, GW-7647 failed to suppress *CYP7A1* expression, whereas it significantly downregulated *GLUT2* (*SLC2A2*) expression in cryopreserved human hepatocytes. It is not clear why *CYP7A1* was not downregulated following PPARα activation in the present study. Marrapodi and Chiang reported that downregulation of *CYP7A1* by Wy14643 is unlikely due to direct regulation by PPARα, but rather indirectly via downregulation of HNF4α (Marrapodi and Chiang, 2000). Notably, their experiments were carried out using HepG2 cells. In the study by Rakhshandehroo et al. (2009). The authors used a different agonist (Wy14643), which may account for the difference. Additionally, differences in culture condition and batch-to-batch variability between cryopreserved human hepatocytes might have contributed to this discrepancy. Differences between previous and our current results may attribute to the level of HNF4α in hepatocytes used and/or the use of different PPARα agonists.

The amino acid catabolism is one of the processes suppressed by PPARα activation. By promoting the proteasomal degradation of hepatocyte nuclear factor 4 alpha (HNF4α), PPARα indirectly downregulates genes encoding amino acid-degrading enzymes, including histidine ammonia-lyase (HAL) and serine dehydratase (SDS) (Rakhshandehroo et al., 2009; Alemán et al., 2013; Tobón-Cornejo et al., 2021). In support of this data, a global transcriptomic analysis of genes differentially regulated by Wy14643 in both mouse and human primary hepatocytes revealed that one of the biological processes that are significantly downregulated include “alpha-amino acid catabolic process” (GO:1901606) and “cellular amino acid catabolic process” (GO:0009063) (Rakhshandehroo et al., 2009). Thus, it is plausible that downregulation of the *NAT2* transcript level by GW-7647 is not a direct result of PPARα activation but of subsequent downregulation of HNF4α. It would be of interest to test if *NAT2* is transcriptionally regulated by HNF4α.

Our previous study showed that *NAT2* mRNA levels in HepG2 cells vary according to the glucose concentration in culture media (Hong et al., 2022). Thus, downregulation of *GLUT2*/*SLC2A2* expression by PPARα activation could potentially lead to a reduction in glucose uptake and indirectly downregulate *NAT2* expression. However, despite the statistical significance, the reduction in *GLUT2* expression was minute, and we question the functional significance of the change.

Although variable NAT2 activity has been linked to physiologically and clinically relevant outcomes (e.g., drug toxicity and dyslipidemia), few studies have investigated its transcriptional regulation. One recent study reported that *NAT2* expression is positively regulated by the vitamin D receptor (VDR) (Zhu et al., 2021). The study showed that treatment with the biologically active form of vitamin D (1,25(OH)_2_D_3_) increases *NAT2* expression and that VDR binds to the *NAT2* promoter in colorectal cancer cells (Zhu et al., 2021). Whether or not *NAT2* is regulated similarly by VDR in the liver remains to be tested.

In summary, we did not observe marked alterations in the *NAT2* transcript level following treatment of synthetic agonists for FXR, PXR, LXR, and PPARα in cryopreserved human hepatocytes. Additional potential transcriptional regulators of hepatic *NAT2* expression, among nuclear receptors, include HNF4α and VDR, which remains to be tested.

## Data Availability

The original contributions presented in the study are included in the article/supplementary material, further inquiries can be directed to the corresponding authors.
